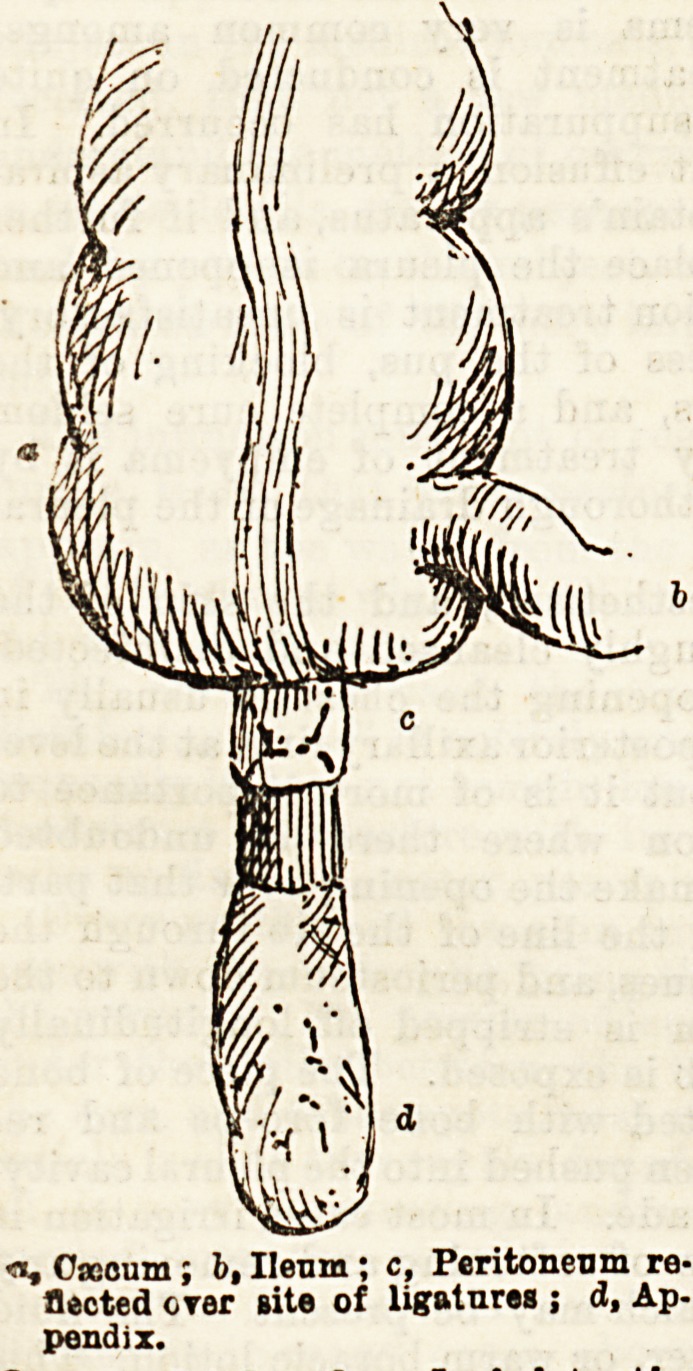# The Treatment of Appendicitis

**Published:** 1893-08-05

**Authors:** H. W. Pigeon

**Affiliations:** Assistant Surgeon to the Hull Royal Infirmary


### The Hospital Clinic.

[*The Editor will be glad to receive offers of co-operation and contributions from members of the profession. All letters should be addressed to* The Editor, The Lodge, Porchester Square, London, W.]

### THE TREATMENT OF APPENDICITIS.

By H. W. Pigeon, M.C. (Cantab.), F.R.C S., Eng., Assistant Surgeon to the Hull Royal Infirmary.

Inflammation in the right iliac fossa, generally called typhlitis, may arise from causes connected with the cæcum, vermiform appendix, or surrounding connective tissue.

The symptoms common to all forms of typhlitis are dull aching pains, tenderness, usually most acute at some point midway between the outer part of Poupart's ligament and the umbilicus, local hardness of the abdominal wall, abdominal distension, colicky pains, vomiting sometimes, constipation, or possibly diarrhœa, temperature raised, and pulse quickened. Recent statistics have shown that the majority of cases of typhlitis occurring in adult men under forty, are due to inflammation of the appendix, from catarrh of the mucous membrane, twisting, the presence of a concretion or foreign body, or from ulceration. The actual cause cannot be diagnosed from symptoms, and certainty is only arrived at by inspection of the parts.

a, Cæcum; b, Ileum; c, Peritoneum reflected over site of ligatures; d, Appendix.

The treatment of appendicitis in the first stage is local, general, and dietetic.

The local treatment consists in giving absolute rest to the inflamed part by keeping the patient in bed, the knees flexed and supported by a pillow. If the pain is acute four leeches are applied, and fomentations of flannel, sponge, or spongio piline; linseed poultices are also useful. The bowels are kept quiet by the administration of pil. opii. gr. ½ every four hours. If flatulence is troublesome the rectum tube is inserted occasionally. No aperients are given. The general treatment consists in the administration of a tonic, such as quiniæ sulph. gr. iiij., acid. sulph. dil. m. x., aq. chlorof. ad. ℥i. Stimulants are given if necessary to maintain strength. The diet is fluid, and such as can be easily digested without leaving a fæcal residue—beef-tea, mutton and chicken broth, and peptonised milk.

Acute appendicitis may subside in a few days with mild treatment. When pain is gone the bowels are relieved by a glycerine or soap and water enema. The absorption of inflammatory induration is hastened by painting the skin over the iliac fossa with tinct. iodi. twice a day for three or four days, or until the skin is too irritated to tolerate it.

The patient is kept in bed for three weeks, when, if any fæcal accumulation is suspected, a dose of ol. ricini ℥ss guarded with tr. opi. ♏xv. is given.

The patient is kept at rest as long as there is any sign of inflammation, as recurrence takes place if active exercise is taken too soon.

*Suppurative Appendicitis.*—The occurrence of suppuration may be suspected if the patient has a rigor, and the temperature is subsequently elevated, or assumes a hectic type. The local signs of pus—redness, œdema, and heat—may be late in appearing, but the pain becomes of an acute throbbing character, and there may be feeling of deep fluctuation.

The introduction of the exploring needle of a hypodermic syringe into the centre of the hardened area may demonstrate the presence of pus. Should no pus be found no harm follows the puncture. In some cases the withdrawal of a little inflammatory exudation determines resolution, owing to the tension being relieved.

If the presence of pus is ascertained, operation is delayed, if the condition of the patient admits, until the barrier of lymph which shuts off the abscess from the general cavity of the peritoneum has had time to consolidate. Some cases admit of a delay for three or four days, but they are carefully watched, and the region of the abscess poulticed.

Operation is not deferred if the temperature keeps high and the pulse rate increases; if there are signs of commencing general peritonitis from leakage or rupture of the abscess; any discoloration or crepitation of the tissues, showing that perforation of the appendix has occurred; or if there is superficial redness and softening, indicating that the abscess is pointing.

Chloroform is preferred to ether, as it is less likely to cause vomiting or bronchial secretion.

Previous to operation the skin over the site of the abscess is brushed over with a solution of iodoform in ether, or powdered iodoform is rubbed on with a little water. An incision about two and a-half inches long is made over the abscess parallel to the outer third of Poupart's ligament, and the peritoneum opened for the full length of the wound. After the pus has escaped the cavity is carefully explored with the forefinger, great care being taken not to break through the limiting lymph barrier. The appendix—thickened, perforated, or gangrenous—is ligatured near its base, cut off, and removed. The ligature is not applied close to the base of the appendix as, if rotten, the tissues are easily cut through by the silk and a second ligature may have to be applied nearer the cæcum. If perforation has occurred, the concretion or foreign body causing it, should be found and removed, if possible, as it does not always escape with the discharges.

The abscess cavity is gently irrigated with carbolic lotion 1 in 60, a large sized drainage tube inserted to the bottom of the cavity, and the wound closed with silkworm gut sutures.

If the general cavity of the peritoneum has been invaded by the pus, the neighbouring intestines are cleaned with sponges, and irrigated with warm boiled water instead of carbolic lotion.

To show the importance of searching for a foreign body or concretion in case of the appendix being found perforated, a case may be mentioned. A woman aged 36, was recently under treatment for a sinus left after the spontaneous rupture of a typhlitic abscess four months previously. On exploration a concretion was found at the bottom of the sinus, and after its removal the opening closed.

*Relapsing Appendicitis.*—Inflammation sometimes recurs, especially when active exercise is resumed too soon after an acute attack has passed off. The patient is usually warned of the possibility of a relapse, so that he may not consider he has been improperly treated. If he is subject to constipation the habitual use of laxatives is advised; and should there be attacks of colicky pain, ol. ricini ℥ss is taken.

Mr. Treves has recently, in the *British Medical Journal*, formulated the conditions in which the removal of the appendix is justifiable.

1. When the attacks of inflammation have been very numerous.
2. When the attacks are increasing in severity.
3. When the last attack has been so severe as to place the patient's life in considerable danger.
4. When constant relapses have reduced the patient to the condition of a chronic invalid, and have rendered him unfit to follow any occupation.
5. When, owing to persistent pain and tenderness during the quiescent period, there is a probability that pus exists in or about the appendix.

It may also be said that the probable risks of an operation in a quiescent state are smaller than the risk of danger in an attack of appendicitis.

Removal of the appendix is undertaken in suitable cases about a fortnight after the symptoms of acute inflammation have subsided. The usual incision is a slightly curved one, 2½ inches long, and two inches internal to the outer part of Poupart's ligament; the centre of the incision lies on a line joining the anterior superior iliac spine and the umbilicus. When the cæcum is exposed the appendix is found by following down the anterior band of longitudinal muscular fibres. The adhesions are separated by the fingers, tough bands being frayed through with the nail. If the appendix is firmly adherent to the bladder, ureter, intestine, or iliac vessels, the adherent portion is cut off, and after stripping off the mucous membrane the remainder is left *in situ*, as Mr. Treves recommends. A medium size silk ligature is placed round the base of the appendix, and, if necessary, another ligature includes the mesentery of the appendix. The peritoneal coat of the appendix is then cut through half an inch from the ligature, and stripped back nearly to the site of the ligature. The appendix is then cut off at the level of the peritoneal reflexion, a sponge being placed underneath to catch any contents that may escape. The exposed portion of mucous membrane is then removed from the stump by a sharp spoon, and the peritoneal cuff drawn back so as to cover in the end of the stump.

Mr. Treves recommends that the edges of the peritoneal cuff should be inverted and brought together with sutures. In one case in which the writer inverted the edges and put in a fine continuous silk suture, suppuration occurred six weeks after the wound was apparently healed, and the suture came away. In another case in which the cuff was simply left covering the stump, and no sutures introduced, the wound healed soundly without any disturbance. No drainage tube is used unless a collection of pus has been found, or the adhesions have been extensive. The wound is closed with silkworm gut sutures passing through the whole thickness of the abdominal walls, and a cyanide gauze dressing applied. The patient is fed by enemata for two days, and treated as after other abdominal operations. The stitches are removed on the tenth day, and the wound supported by strips of strapping. The patient is kept in bed for three weeks, and an abdominal belt with pad supplied before he is allowed to return home.

## Figures and Tables

**Figure f1:**